# Supplementary material for: The causal relationship between the human gut microbiota and pyogenic arthritis: a Mendelian randomization study
Source: Front Cell Infect Microbiol. 2024 Nov 26;14:1452480. doi: 10.3389/fcimb.2024.1452480 (PMC11629706; doi:10.3389/fcimb.2024.1452480)
Supplement: Supplementary file 1 [file DataSheet1.zip › mendelian test/ebi-a-GCST90027736.csv_leave_one_out.pdf]

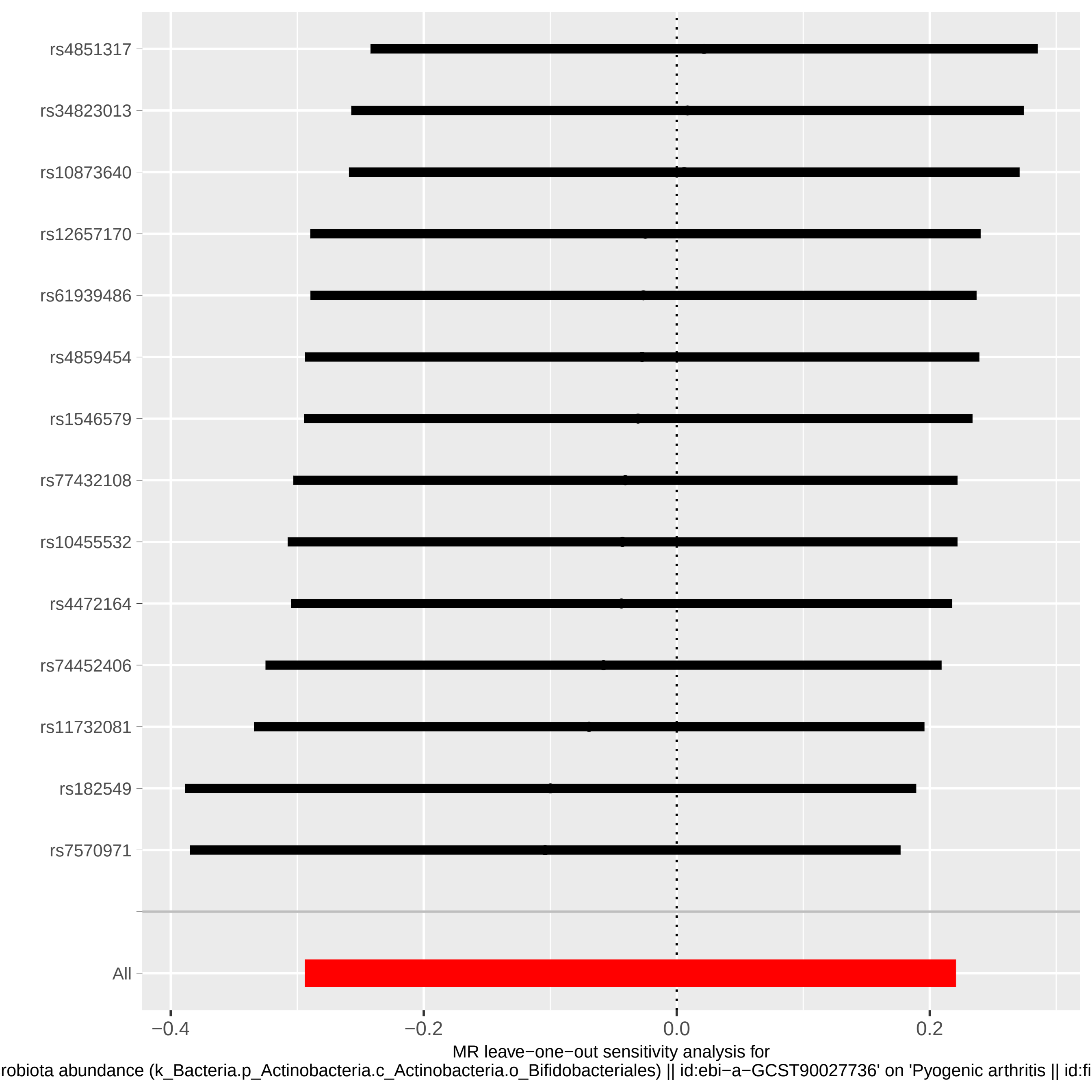
rs4851317
rs34823013
rs10873640
rs12657170
rs61939486
rs4859454
rs1546579
rs77432108
rs10455532
rs4472164
rs74452406
rs11732081
rs182549
rs7570971
All
−0.4
−0.2
0.0
0.2
MR leave−one−out sensitivity analysis for
robiota abundance (k_Bacteria.p_Actinobacteria.c_Actinobacteria.o_Bifidobacteriales) || id:ebi−a−GCST90027736' on 'Pyogenic arthritis || id:fi
